# Growth hormone restrains inflammatory activity of microglia/macrophages in CNS autoimmunity

**DOI:** 10.3389/fncel.2026.1828468

**Published:** 2026-08-04

**Authors:** Anica Zivkovic, Milica Lazarević, Ivana Stevanovic, Danijela Laketa, Đorđe Miljković, Irena Lavrnja

**Affiliations:** 1 Department of Neurobiology, Institute for Biological Research “Sinisa Stankovic”, National Institute of Republic of Serbia, University of Belgrade, Belgrade, Serbia; 2 Department of Immunology, Institute for Biological Research “Sinisa Stankovic”, National Institute of Republic of Serbia, University of Belgrade, Belgrade, Serbia; 3 Medical Faculty of Military Medical Academy, University of Defense, Belgrade, Serbia; 4 Department for General Physiology and Biophysics, Faculty of Biology, University of Belgrade, Belgrade, Serbia

**Keywords:** disease-modifying therapy, EAE, growth hormone, macrophage, microglia, morphometry

## Abstract

Growth hormone (GH) has been implicated in immune regulation, but its effects on microglia/macrophages during neuroinflammation remain insufficiently characterized. Here, we investigated the impact of GH on microglia/macrophages activation using complementary *in vitro* and *in vivo* approaches. In LPS-stimulated BV2 microglia, GH induced anti-inflammatory and antioxidant responses, accompanied by morphological features consistent with reduced cellular activation. *In vivo*, GH administration in rats with experimental autoimmune encephalomyelitis (EAE) delayed disease onset and significantly reduced disease duration, severity, and cumulative clinical score. GH also affected peripheral immune responses, as evidenced by decreased production of the pro-inflammatory cytokines interferon-γ and interleukin-17 by lymph node cells draining the site of the immunization. However, the major effect of GH was observed in the central nervous system, where GH profoundly altered the inflammatory amoeboid morphology of microglia/macrophages typically observed at the EAE peak. Quantitative morphometric, skeleton, and fractal analyses of microglia/macrophages demonstrated reduced process complexity, decreased cell area and perimeter, increased circularity and solidity, reduced fractal dimension and lacunarity, and diminished branching parameters, accompanied by increased iNOS expression at the EAE peak. GH treatment prevented these changes, restoring microglia/macrophages morphological complexity and attenuating pro-inflammatory features. Collectively, these findings indicate that GH modulates neuroinflammatory responses by regulating the microglia/macrophages activation state, supporting a more ramified, surveillant microglia/macrophages phenotype. This positions GH as a disease phase-sensitive regulator of neuroinflammatory processes relevant to autoimmune disorders of the central nervous system.

## Graphical abstract

**Figure:**
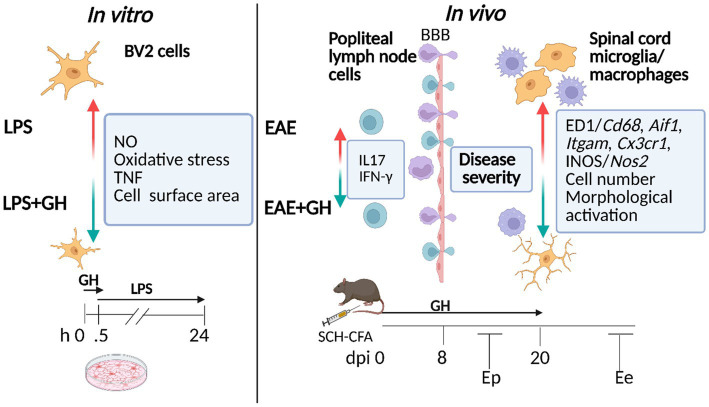
Growth hormone as neuroimmune modulator *in vitro* and *in vivo*.

## Introduction

Multiple sclerosis (MS) is a chronic, inflammatory disease of the central nervous system (CNS). It is marked by persistent neuroinflammation that leads to demyelination and progressive neurodegeneration. As a consequence, MS frequently results in long-term disability in working-age adults, imposing a substantial socioeconomic burden on modern societies (Bjelobaba et al., 2017). Most of the data on the underlying pathological mechanisms have been obtained from the commonly used animal model of MS, experimental autoimmune encephalomyelitis (EAE) (Bjelobaba et al., 2018). Autoimmune reactivity against CNS structures is a hallmark of MS and EAE pathogenesis. Encephalitogenic CD4^+^ T lymphocytes, particularly T helper (Th)1 and Th17 subsets, are widely recognized as key factors in MS/EAE pathogenesis (Sospedra and Martin, 2016). These cells, initially activated in the periphery, infiltrate the CNS where they encounter CNS antigens presented by resident macrophages and microglia (Van Hove et al., 2025). In response to reactivation Th1 and Th17 cells release pro-inflammatory cytokines, such as IFN-γ and IL-17, respectively. Microglia/macrophages also produce large amounts of reactive oxygen species (ROS), nitric oxide (NO) and inflammatory cytokines, such as tumor necrosis factor (TNF), which together with cytokines produced by T cells, establish a full-blown inflammation within the CNS (Sospedra and Martin, 2016; Silvin and Qian, 2023). This uncontrolled inflammation drives the demyelination and neuronal damage characteristic of MS (Lee, 2018; Vasileiadis et al., 2018). During neuroinflammation, microglial activation is associated with distinct morphological changes. Activated microglia adopt an amoeboid shape, upregulate activation markers, and secrete various pro-inflammatory substances, such as NO, ROS, TNF, and other inflammatory cytokines (Jiang et al., 2014). Inflammatory microglia is characterized by reduced branching, decreased endpoints, and lower overall complexity, whereas anti-inflammatory or reparative microglia display highly ramified processes and complex branching patterns, reflecting their tissue-protective roles (Plastini et al., 2020). Therefore, morphometric and fractal analyses of microglia are powerful tools to delineate activation states and functional phenotypes in CNS disease models (Fernández-Arjona et al., 2017).

Growth hormone (GH, somatotropin), produced by the adenohypophysis, exerts pleiotropic effects on growth, metabolism, immune regulation, and CNS homeostasis (Bioletto et al., 2025; Hattori, 2009; Soler Palacios and Nieto, 2020). Consequently, recombinant human GH therapy could offer new therapeutic opportunities for neuroinflammatory diseases in addition to its primary use in treating GH deficiency (Ranke and Wit, 2018). GH acts directly via the GH receptor (GHR) or indirectly through IGF-1, promoting neuroprotection, synaptic plasticity, and proliferation of neural progenitors (Pathipati et al., 2009; Feeney et al., 2017; Sanchez-Bezanilla et al., 2020). GHR is expressed on immune cells, including T and B lymphocytes, monocytes, and macrophages, which can also produce GH, highlighting its autocrine and paracrine roles in immune modulation (Hattori et al., 2001; Soler Palacios and Nieto, 2020; Wasinski et al., 2023). Previous studies have shown that GH can exert context-dependent effects on myeloid cells, inducing either inflammatory or anti-inflammatory states, downregulating the NLRP3 inflammasome, and promoting tissue repair in inflammatory conditions (Schneider et al., 2019; Soler Palacios et al., 2023; Spadaro et al., 2016; Villares et al., 2018). GHR is also expressed on microglial cells (Tavares et al., 2024), yet the effects of GH on microglia/macrophages activation during neuroinflammation remain largely unexplored.

The present study investigates the impact of inflammation on activation of microglia/ macrophages, with a particular emphasis on the immunomodulatory effects of GH. We first confirmed the anti-inflammatory and antioxidant actions of GH in LPS-activated BV-2 microglial cells. Next, we evaluated the pathogenesis of EAE and the associated neuroinflammatory response throughout disease progression, with special attention to microglia/macrophages dynamics during different disease stages and following GH treatment. Finally, we assessed the effects of GH on EAE onset and progression, focusing specifically on microglia/macrophages morphology, quantitative morphometric parameters, and functional phenotypes, as key determinants of neuroinflammatory outcome. To our knowledge, this is the first study to examine GH treatment in EAE with a dedicated and comprehensive analysis of microglia/macrophages phenotypic modulation, highlighting microglia/macrophages as a key cellular target of GH-mediated neuroprotection.

## Materials and methods

### Cell culture

BV2 murine microglial cells (Elabscience Biotechnology Inc., Houston, TX, USA; Cat. No. EP-CL-0493) were maintained in RPMI 1640 medium supplemented with 10% heat-inactivated fetal bovine serum (FBS), 100 U/mL penicillin, and 100 μg/mL streptomycin (all from Gibco, Thermo Fisher Scientific, Waltham, MA, USA). Cultures were incubated at 37 °C in a humidified atmosphere containing 5% CO₂. When the cells reached 80–90% confluence, they were detached using 0.1% trypsin–EDTA solution (Sigma-Aldrich, St. Louis, MO, USA) and reseeded for subsequent experiments. The BV2 microglia used in this study were divided into four experimental groups based on the applied treatment conditions. The untreated BV2 cells served as the control group. The GH group was treated with 100 μM recombinant human growth hormone (GH) (Norditropin® NordiLet® by NovoNordisk Health Care AG, Zürich Switzerland). The LPS group was treated with 1 μg/mL Lipopolysaccharide (LPS from *Escherichia coli* serotype 026:B6; Sigma–Aldrich Labware, Munich, Germany) for 24 h and LPS + GH group—cells were pretreated with 100 μM GH for 30 min and then stimulated with 1 μg/mL LPS for 24 h.

### Cell viability assay

To assess cell viability, we used the MTT assay as previously described (Milosevic et al., 2022). Briefly, cells were plated in 96-well plates at a density of 1 × 10^4^ cells per well and incubated for 24 h. Subsequently, the cells were treated with various concentrations of GH for a further 24 h in the presence or absence of 1 μg/mL LPS. The absorbance was measured at 492 nm using a microplate reader (Synergy H1M, BioTek Instruments Inc., Winooski, VT, USA). The results are expressed as % of mean optical density (OD 492 nm) relative to control ± SEM, from three independent experiments performed in triplicate.

### Measurement of nitric oxide (NO) production

The colorimetric Griess method was used to indirectly determine NO released in cell culture supernatants by measuring nitrite, a primary and stable product of NO, as previously described (Milosevic et al., 2022). Briefly, BV-2 microglial cells were seeded into 24-well plates (density of 5 × 10^4^ cells per well) and subjected to the described treatment conditions. Following treatment, the cell-free culture medium was collected, and analyzed for NO concentration by measuring nitrite and nitrate levels. Spectrophotometric measurements were performed at 570 nm (Synergy H1M). The results are presented as the mean nitrite concentration (μM) ± SEM, based on three independent determinations.

### F-actin staining

Cell morphology was assessed using fluorescence microscopy of the cytoskeleton, as previously described (Bozic et al., 2015a). BV-2 microglial cells were seeded at a density of 8 × 10^4^ onto glass coverslips (25 mm diameter) placed in 35 mm culture dishes (Sarstedt, Newton, NC, USA). Filamentous F-actin was labeled with Alexa Fluor 555–conjugated phalloidin (Invitrogen, Carlsbad, CA, USA; 1:50 dilution in PBS, 30 min incubation). Following PBS washes, nuclei were counterstained with Hoechst 33342 (5 μg/mL; Life Technologies, Invitrogen, Carlsbad, CA, USA). Coverslips were mounted using Mowiol medium (Calbiochem, Darmstadt, Germany), and images were acquired with a Zeiss Axiovert fluorescence microscope (Zeiss, Jena, Germany). Images of phalloidin-stained cells were analyzed to determine average cell surface area in each experimental group using AxioVisionRel 4.6 software (Zeiss, Jena, Germany). For each coverslip, five regions of interest (138 × 104 μm^2^) were evaluated, with three coverslips examined per group.

### Assessment of oxidative stress markers and antioxidant enzyme activities

Oxidative stress markers and antioxidant enzyme activities were performed as previously described (Bozic et al., 2015b). Briefly, superoxide anion (·O₂^−^) production was determined by measuring the reduction of nitroblue tetrazolium (NBT) to monoformazan in an alkaline nitrogen-saturated medium. Absorbance was recorded at 550 nm, and results were expressed as μmol/mg protein (mean ± SEM). Malondialdehyde (MDA) levels were assessed by the thiobarbituric acid (TBA) reaction. Samples were incubated with TBA reagent (15% trichloroacetic acid and 0.375% TBA) at 95 °C (pH 3.5), and absorbance was measured at 532 nm. Results are presented as mol/mg protein (mean ± SEM). Total glutathione content was measured using the DTNB–GSSG reductase recycling assay. Formation of 5-thio-2-nitrobenzoic acid was monitored at 412 nm and expressed as mol/mg protein (mean ± SEM). Catalase (CAT) activity was determined spectrophotometrically at 405 nm based on the formation of a colored complex between ammonium molybdate and residual H₂O₂. One unit represents μmol H₂O₂ decomposed per minute. Results are expressed as U/mg protein (mean ± SEM). Glutathione peroxidase (GPx) activity was assessed indirectly by measuring NADPH oxidation at 340 nm and expressed as mU/mg protein. Glutathione reductase (GR) activity was determined by monitoring NADPH oxidation during the reduction of GSSG to GSH and expressed as mU/mg protein. All measurements were performed in duplicate in at least three independent experiments.

### Animals

All experimental procedures were conducted in accordance with national regulations governing the use of animals in research and adhered to Directive 2010/63/EU of the Council of the European Communities. The work also complied with the Law on Animal Protection of the Republic of Serbia (Official Gazette of the Republic of Serbia, No. 41/09). Approval for the study was granted by the Veterinary Administration of the Ministry of Agriculture, Forestry and Water Management, Republic of Serbia, which issued the authorization under permit number 01–1594/2024. Given the higher prevalence of MS in females and well-established sex-dependent differences in immune and glial responses, we used female animals as a clinically relevant EAE model (Alvarez-Sanchez and Dunn, 2023). Female Dark Agouti (DA) (RRID:RGD_21409748) rats were used. DA rats were bred at approximately 2 months of age and housed at the local animal facility in groups of 4 animals per cage on a 12:12-h light:dark cycle with ad libitum access to food and water.

### EAE induction and therapeutic interventions

EAE was induced as previously described (Zivkovic et al., 2024). Recombinant human GH was administered at a dose of 200 μg/kg body weight i.p. daily for 20 consecutive days from the day of immunization (EAE + GH) or until euthanasia at the 8 day post immunization (d.p.i) and/or peak and end of symptoms. Non-induced age-matched littermate females were used as controls (C). The animals were observed daily during the experiment and examined for neurological deficits. Clinical severity was graded according to a predetermined scale based on criteria: 0, asymptomatic; 1, tail atony—complete loss of tail tone; 2, hind limb paraplegia; 3, complete hind limb paralysis; 4, hind limb paralysis/moribund; and 5, death. At a score of 3, rats were provided with food and water on the cage floor to facilitate access, while the humane endpoint was set at a score of 4 for two consecutive days. In addition, the rats’ body weight was measured daily.

The severity of the ongoing disease was assessed by evaluating and calculating the following parameters. Disease onset: the average day when the first symptom appeared. Disease duration: the average of the total number of days an animal exhibited any symptoms of EAE. Paralysis duration: the average total number of days an animal experienced paralysis. Maximal clinical score: the average of the highest clinical scores recorded for each animal within the group. Cumulative disease index: the average of the total symptom scores an animal accumulated throughout the disease course.

### Tissue preparation

The rats were euthanized in a CO_2_ chamber with a controlled displacement rate of approximately 30% of the chamber volume per minute, ensuring gradual loss of consciousness. EAE rats were sacrificed at three time points of EAE: onset at 8 d.p.i., peak at 14 d.p.i, and at the end of EAE at 28 d.p.i. Blood was collected by cardiac puncture into plain tubes for serum separation. Samples were centrifuged at 7500 × *g* for 10 min at 4 °C, and the resulting serum was carefully transferred into fresh tubes. Aliquots were stored at −80 °C until further analysis to prevent repeated freeze–thaw cycles.

Popliteal lymph node cells (PLNC) were obtained from immunized rats at 8 d.p.i and inguinal lymph node cells from non-immunized rats as previously described (Lazarević et al., 2020). To obtain cell culture supernatants, PLNC were seeded at 5 × 106 /mL/well and stimulated with 10 μg/mL MOG for 48 h.

As the observed disease symptoms primarily affected the lower extremities, cellular analyses were performed on the lumbar spinal cord. For gene expression studies, the lumbar part of the spinal cords (*n* = 6/group) was manually dissected at the respective time points after perfusion with 50 mL ice-cold phosphate buffer, then immersed in RNAlater® RNA Stabilization Solution (Ambion™, Applied Biosystems by Thermo Fisher Scientific, Waltham, MA) and stored at −80 °C until further processing.

For immunohistochemical studies, all groups were transcardially perfused with 50 mL ice-cold phosphate buffer and the spinal cords were placed in 4% PFA for 24 h. The tissue was then immersed in ascending sucrose solution (10–30%), immersed in 2 methyl-butane, embedded in Tissue Tek (OCT, Sakura, Finetek, USA) and frozen at −80 °C. The spinal cords were cryosectioned (16 μm) at optimal sectioning temperature, collected on gelatin slides and stored at −20 °C until use. For morphometric studies, the spinal cords were cryosectioned at 50 μm.

### Biochemical analysis

Serum biochemical parameters were measured using the Mindray BS-240 Vet automated analyzer (Mindray, China), based on spectrophotometric and turbidimetric methods. All analyses were performed using commercial reagents from BioSystems (Barcelona, Spain), following the manufacturer’s instructions. Serum samples were thawed and vortexed prior to analysis, and processed under controlled conditions. Calibration and quality control were performed according to the manufacturers’ protocols.

### ELISA tests

PLNC cell-free culture supernatants were obtained by centrifugation at 500 g for 3 min and IFN-γ and IL-17 concentrations in these supernatans were measured by the sandwich ELISA method using appropriate capture and detection antibodies for IFN-γ and IL-17 (Thermo Fisher Scientific, Waltham, MA). The lower limit of detection was 30 pg/mL, and the upper limit of detection was 10 ng/mL. Standard curves were generated based on the known concentrations of recombinant IFN-γ, and IL-17 (Peprotech, Rocky Hill, NJ).

The supernatants from both treated and control BV2 cell cultures were collected for analysis. TNF levels were quantified using an ELISA kit (eBioscience by Thermo Fisher Scientific, Waltham, MA, USA), following the protocol provided by the manufacturer. The lower limit of detection was 25 pg/mL, while the upper limit of detection was 20 ng/mL. Standard curves were generated based on the known concentrations of recombinant TNF (Peprotech, Rocky Hill, NJ). Absorbance readings for each well were recorded at 450 nm using an enzyme plate analyzer (Synergy H1M).

In addition, serum samples from all experimental groups were analyzed for these cytokines.

### Quantitative real-time PCR

Total RNA was extracted from PLNC after 48 h restimulation with MOG and the spinal cord of all experimental animal groups. Isolation was performed using the RNeasy Mini Kit (QIAGEN, Hilden, Germany). RNA (1 μg) was reverse transcribed using a High Capacity cDNA Reverse Transcription Kit (Applied Biosystems by Thermo Fisher Scientific). Quantitative real-time PCR (qPCR) analysis was performed with Power SYBR_TM_ Green PCR Master Mix (Applied Biosystems by Thermo Fisher Scientific) and primers designed on specific targets using a QuantStudio™ 3 Real-Time PCR System (Applied Biosystems by Thermo Fisher Scientific). Relative mRNA expression (Table 1) was normalized to *Gapdh* as the reference gene and calculated using the 2^−ΔCt^ method. Results are expressed as mean ±SEM and were obtained from 6 replicates per group from 2 independent experiments.

**Table 1:** Primer list.

Gene symbol	Forward primer sequence	Reverse primer sequence	Accession number
*Gapdh*	TGGACCTCATGGCCTACAT	GGATGGAATTGTGAGGGAGA	NM_017008.4
*IFNɣ*	TTACTGCCAAGGCACACTCAT	GTGTTACCGTCCTTTTGCCA	NM_138880.2
*IL17*	GTTCAGTGTGTCCAAACGCC	AGGGTGAAGTGGAACGGTTG	NM_001106897.1
*Aif1*	CCAGCGTCTGAGGAGCTATG	CGTCTTGAAGGCCTCCAGTT	NM_017196.3
*Cd68*	TGTGTGTCTGACCTTGCTGG	AAGGATGGCAGAAGAGTGGC	NM_001031638.1
*Nos2*	ACACAGTGTCGCTGGTTTGA	AACTCTGCTGTTCTCCGTGG	NM_012611.3
*Arg1*	CTGTGGTAGCAGAGACCCAGA	GGTTGTCAGCGGAGTGTTGA	NM_017134.3
*Mrc1*	CAGACCCACTGACTGGCATT	GCTCGTGAATCTCCGTGACA	NM_001106123.2
*Itgam*	GACTCCGCATTTGCCCTACT	TGCCCACAATGAGTGGTACAG	NM_012711.1
*Cx3cr1*	TTCTTCCTCTTCTGGACGCCT	TGAGGCAGCAGTGGCTAAAC	NM_133534.1

### Immunohistochemistry

To perform immunohistochemistry, sections were rehydrated in PBS, then incubated for 30 min in citrate buffer in water bath warmed to 80 °C for antigen retrieval. For morphometric studies sections were incubated in 0.1% Triton X-100 (Sigma-Aldrich, St. Louis, MO, USA) for 20 min. After this, sections were washed in PBS and nonspecific binding sites were blocked in 10% normal donkey serum or 5% BSA for 30 min. The sections were then incubated overnight (at 4 °C) with primary antibodies (Table 2).

**Table 2:** Antibody list.

Antibody	Source and type	Dilution	Manufacturer
IBA1	Goat, polyclonal	1:400	Abcam, ab5076
	Rabbit, monoclonal	1:200	Abcam, ab178846
iNOS	Rabbit, polyclonal	1:200	Abcam, ab15323
ARG1	Rabbit, polyclonal	1:200	Novus Biologicals, NBP1-32731
Anti-CD68 antibody [ED1]	mouse monoclonal	1:100	Abcam, ab31630
Anti-Myelin Basic Protein antibody [EPR21188]—Oligodendrocyte Marker	Rabbit, polyclonal	1:200	Abcam, ab218011
Anti-goat IgG AlexaFluor 568	Donkey	1:200	Invitrogen, A11057
Anti-rabbit IgG AlexaFluor 488	Donkey	1:200	Invitrogen, A21206
Anti-mouse IgG AlexaFluor 568	Donkey	1:200	Invitrogen, A10037

After incubation with the primary antibody and washing with PBS, the sections were incubated with the appropriate secondary antibody. For morphometric analysis (50 μm sections), sections were incubated with an Alexa Fluor 488–conjugated secondary antibody. Sections were then washed again in PBS and mounted with Mowiol embedding medium (Merck Millipore, Darmstadt, Germany). To ensure specificity of staining, negative controls (without primary antibodies) were performed in parallel.

For morphometric studies, high-resolution fluorescence images of IBA1-positive microglia/macrophages were acquired from 50 μm spinal cord sections. Images were captured in single confocal planes using a confocal laser scanning microscope (LSM 510, Zeiss) at 40× magnification.

For 16 μm spinal cord sections, imaging was performed using a Zeiss Axiovert microscope equipped with ZEN software (Carl Zeiss GmbH, Vienna, Austria). To ensure consistency, identical microscope settings were maintained across all experimental groups. On these sections, morphometric and densitometric analysis of ED1^+^ cells were performed. Measurements included Integrated Density (IntDen), Corrected Total Cellular Fluorescence (CTCF), and the percentage of immunoreactive area (%Area). This quantitative image analysis was performed using ImageJ. Images were converted to 8-bit grayscale, after which a uniform threshold was applied to all images to define the ROI. Measurements were then obtained using the “Measure” function. The percentage of area (% area) was calculated as the proportion of threshold-positive area relative to the total analyzed field. Integrated density (IntDen) was defined as the sum of pixel intensity values within the ROI. Corrected total cell fluorescence (CTCF) was calculated using the formula: CTCF = Integrated Density − (Area of ROI × Mean background fluorescence), where background intensity was measured in regions without specific signal. To evaluate image quality and intensity profiles, the fraction of saturated pixels was calculated for each image. Across all analyzed channels/images, the maximum intensity values remained strictly bounded between 71 and 90 on an 8-bit scale (0–255), indicating zero overexposure (0% saturation at the maximum intensity level). Regarding the minimum intensity level (value 0), the typical fraction of saturated pixels in the dark background (shadow clipping) was found to be 0.0144 (1.44%). The majority of the images exhibited negligible background saturation (ranging from 0 to 0.03%), while only two specific frames showed a higher background fraction of 9.3 and 5.8%, respectively. This confirms that the dynamic range of the signal was properly captured without significant loss of intensity information.

In addition, on 16 μm spinal cord sections, colocalization between fluorescent signals was quantified using Pearson’s correlation coefficient (*P*) and Manders’ overlap coefficients (M1 and M2). Images were acquired under identical microscope settings for all experimental groups and analyzed using *ImageJ/Fiji* (NIH, USA) with the Coloc2 plugin. Prior to analysis, ROIs were manually defined to include only relevant tissue areas, and background fluorescence was minimized using uniform thresholding applied equally across all samples.

Pearson’s correlation coefficient was used to assess the linear correlation between the intensity distributions of the two fluorescence channels, with values ranging from −1 (complete negative correlation) to +1 (complete positive correlation). Manders’ overlap coefficients (M1 and M2) were calculated to determine the fraction of one fluorophore signal overlapping with the other, independent of signal intensity, with values ranging from 0 (no overlap) to 1 (complete overlap). Colocalization values were calculated for each image, and mean values were used for statistical analysis.

For quantification of IBA1^+^ microglia/macrophages (50 μm spinal cord sections), micrographs were acquired under identical exposure settings to allow reliable comparison between experimental groups. For each animal, five non-overlapping regions of interest (ROIs) were selected from the white matter of the spinal cord at defined and reproducible anatomical locations, including two lateral white matter regions, two ventral regions on either side of the blood vessels, and one dorsal white matter region. Only IBA1-positive cells with clearly defined processes, located entirely within the predefined ROIs were included. Cells positioned at the edges of the field of view or regions of interest were excluded to avoid partial reconstruction bias. To further reduce bias, cell selection was performed in a systematic and blinded manner across standardized anatomical locations. Cell counting was performed using ImageJ software (NIH, Bethesda, MD, USA). Images were first converted to 8-bit grayscale and the “Multi-point” function was used to count positively stained cells and these results were then normalized on mm^2^ of ROI surface. Counts from five ROIs per section and three sections per animal were averaged to obtain a representative value for each animal. Data are presented as the mean ± SEM from three independent animals per group.

### Microglia/macrophage morphology analysis

Quantitative characteristics of microglia/macrophage morphology, including soma dimensions, process elongation, branching patterns, and fractal properties, provide robust indicators of microglia/macrophage activation and functional status, reflecting the close relationship between cellular structure and function in CNS pathology (Karperien and Jelinek, 2015; Fernández-Arjona et al., 2017; Green et al., 2022). Analyses were performed on microglia/macrophage from six animals, with five spinal cord sections (50 μm sections) per animal and four to five cells analyzed per section, resulting in a total of 120–150 microglia/macrophage cells per group. Microglia/macrophage morphology was assessed using Fiji (Fiji Is Just ImageJ, ImageJ 1.54p; version 21.0.7, NIH, USA) and specialized plugins to capture complementary aspects of cell structure. For all analyses, individual microglia/macrophage cells were manually selected to avoid overlapping cells and truncated processes. Images were adjusted as previously described (Young and Morrison, 2018). Briefly, to ensure comparability across analyses, all images underwent the same preprocessing workflow. They were converted to 8-bit grayscale, brightness and contrast were adjusted when necessary, and filters were applied sequentially: unsharp mask (pixel radius 3, mask weight 0.6), despeckle, threshold adjustment, binary close, despeckle, and removal of outliers (pixel radius 2, threshold 50). The processed images were then saved for further quantification. Simple shape descriptors such as area (μm^2^), perimeter length (μm), circularity, roundness and solidity were characterized using Analyze Particles. Higher circularity, roundness and solidity values indicated an amoeboid, activated phenotype, whereas lower values reflected increased ramification and process complexity. Skeleton analysis provided detailed measures of microglia/macrophage process architecture. Binarized cell masks were skeletonized using the Skeletonize (2D/3D) plugin and analyzed with AnalyzeSkeleton. For each cell, total process length was calculated as the sum of all branches, endpoints represented terminal tips, and branch number reflected network complexity (Green et al., 2022). Fractal analysis was performed using the FracLac plugin, applying the box-counting method to binarized cell outlines. Parameters obtained included fractal dimension, lacunarity, density, and span ratio. In the Grid Design settings, the number of grid orientations (Num G) was set to 4. Within the Graphics options, the Metrics box was checked to enable analysis of the convex hull and bounding circle of each cell. The scanning procedure generated three output windows: Hull and Circle Results, Box Count Summary File, and Scan Types. From the Hull and Circle Results window, relevant parameters such as density and span ratio were extracted, while fractal dimension and lacunarity values were obtained from the Box Count Summary window. To ensure statistical validity and avoid pseudoreplication, all analyses were performed using animal-based averaging. Specifically, individual cell measurements were first averaged within each animal to generate a single value per biological replicate. These animal-specific means were subsequently used as the independent data points for all statistical comparisons.

## Statistical analysis

All statistical analyses were performed using GraphPad software (RRID: SCR_002798, GraphPad Prism version 8 for Windows, GraphPad Software, La Jolla, CA, USA). The significance of differences between groups was assessed using one-way ANOVA followed by appropriate *post hoc* multiple comparison tests when more than two groups were analyzed and data passed normality assumptions. For comparisons between two groups, Welch’s unpaired *t*-test was applied. In cases where data did not meet normality criteria, non-parametric tests (Kruskal–Wallis test followed by Dunn’s post hoc test, or Mann–Whitney *U* test) were used as appropriate. For morphometric analyses, multiple cells were quantified per section and per animal (*n* = 6 animals per group). Quantitative data were expressed as mean ± SEM, and values of *p* ≥ 0.05 were considered statistically significant.

## Results

### GH reduces LPS-induced inflammatory activity of BV2 cells

To test the effects of GH on BV-2 cell viability, these cells were exposed to different concentrations of GH in the presence or absence of LPS. GH applied at concentrations up to 100 μM did not affect the viability of either LPS-stimulated or unstimulated BV2 cells (Figure 1A). Therefore, 100 μM GH was used in subsequent experiments. Next, we assessed the effect of GH on NO and TNF production in BV2 cells. GH did not significantly influence NO and TNF production in BV2 cells that were not stimulated with LPS, while it significantly reduced the ability of LPS-stimulated BV2 cells to produce these inflammatory mediators (Figures 1B,C).

**Figure 1:**
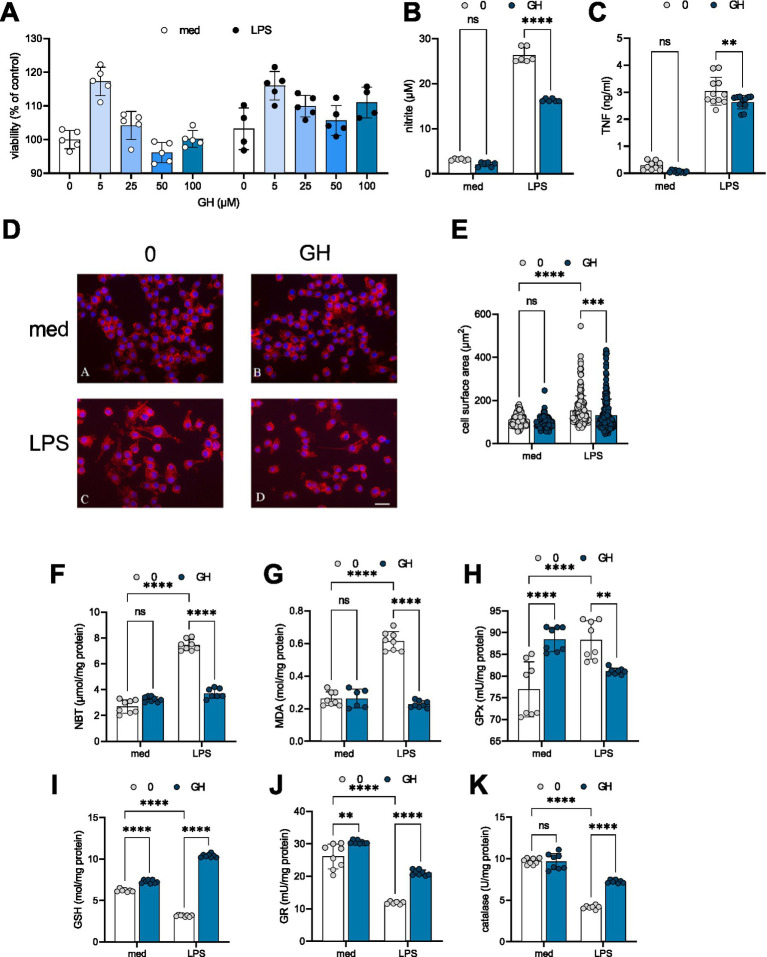
Effects of GH on BV2 cells. Cell viability was assessed in non-stimulated (med) or LPS-stimulated BV2 microglial cells exposed to the indicated levels of GH for 24 h, using MTT test **(A)**. Mean value of A_492_ in GH-untreated BV2 cells (0) was set as 100%. Non-stimulated or LPS-stimulated BV2 cells were cultivated in the absence (0) or presence of 100 μM GH for 24 h **(B–K)**. Nitrites were detected by Griess reaction **(B)**, while TNF levels in cell culture supernatants were determined by ELISA **(C)**. Representative fluorescence microscopy images of BV2 cells stained with F-actin (red) and Hoechst 33342 (blue) were made **(D)**. Images were acquired using fluorescence microscopy and analyzed with Zeiss ZEN 3.8 software. Scale bar: [20 μm]. Quantification of BV2 cell surface area based on F-actin/Hoechst fluorescence microscopy was performed **(E)**. Production of superoxide anion was measured by NBT test **(F)**, lipid peroxidation by MDA test **(G)**, level of GPx using **(H)**, catalase activity using **(I)**, GSH level using **(J)**, and GR activity by **(K)**. Bar graphs represent mean + SD (*n* = 3, **A–C**; *n* = 200 cells, **E**; *n* = 8, **F–K**). ^*^p < 0.05, ^**^p < 0.01, ^****^p < 0.001, ^***^p < 0.0001, ns, not significant.

To further assess the effect of GH on BV2, cell morphology analysis was performed. Non-stimulated BV2 cells exhibited a rounded, amoeboid morphology with a uniform, punctate pattern of F-actin distribution (Figure 1D). LPS stimulation caused dramatic morphological changes in BV2 cells, which appeared enlarged with extended processes ending in distinct microvilli (Figure 1D). While GH did not change morphology of non-stimulated BV2 cells, it counteracted morphological shift induced by LPS (Figure 1D). These observations were supported by quantitative analysis of cell surface area (Figure 1E).

To determine whether GH exerts antioxidative effects during microglia activation, we evaluated superoxide anion production, lipid peroxidation, GPx catalase, and GR activity, and GSH levels. LPS stimulation increased superoxide anion generation, lipid peroxidation and GPx activity, while GH counteracted the effects of LPS on BV2 cells (Figures 1F–H). On the contrary, LPS stimulation decreased catalase and GR activity, as well as GSH levels, and GH was able to counteract these effects of LPS on BV2 cells, as well (Figures 1I–K).

### GH ameliorates EAE

To investigate the potential therapeutic effect of GH on the development and severity of EAE, immunized animals were treated with GH from the day of the immunization. The treatment delayed the onset of EAE and did not reduce body weight (Figures 2A,B). Besides the delay in the disease onset (Figure 2C), treatment with GH also reduced the disease duration (Figure 2D), the period of paralysis (Figure 2E), and the cumulative clinical score (Figure 2F). No significant effect of GH treatment on the maximal c.s. was observed (Figure 2G). Mirroring these clinical improvements, histopathological evaluation using hematoxylin staining confirmed that GH treatment at peak of disease noticeably diminished the infiltration of inflammatory cells into the spinal cord parenchyma (Supplementary Figure 1 – Data Sheet 1).

**Figure 2:**
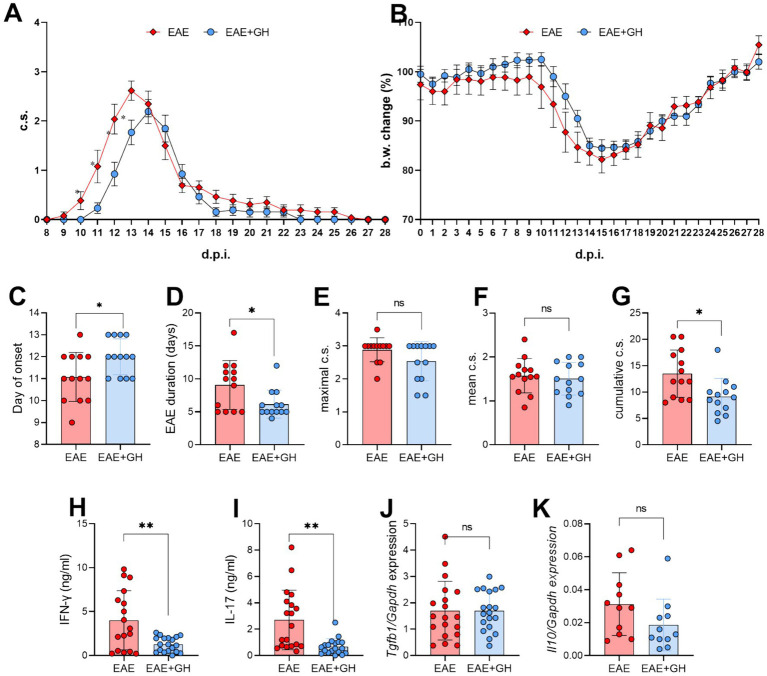
Effects of GH on EAE. EAE was induced in DA rats using SCH + CFA. GH was administered daily for 20 days following immunization. Clinical scores (c.s.) and body weight (b.w.) were assessed daily for 28 days post-immunization **(A,B)**. Onset, duration of disease, duration of paralysis, cumulative c.s., and maximal c.s. were determined **(C–G)**. PLNC were obtained on 8 d.p.i. and stimulated with MOG (10 μg/mL). Subsequently, levels of IFN-γ **(H)** and IL-17 **(I)** were determined by ELISA, while expression of mRNA for IL-10 **(J)**, and TGF-β **(K)** were determined using qPCR. Data are presented as mean ± SEM from (**A,B**, *n* = 13) or as mean + SD (**C–G**, *n* = 13; **H–K**, *n* = 19). ^*^p < 0.05, ^**^p < 0.01, ^****^p < 0.001, ^***^p < 0.0001, ns – not significant.

To explore the effects of GH treatment for EAE on the peripheral immune response, PLNC were collected from rats at 8 d.p.i. The total number of PLNC did not differ significantly between EAE and EAE-GH group (50.2 ± 4.7 × 10^6^ vs. 53.9 ± 4.9 × 10^6^, respectively). GH treatment significantly reduced the secretion of key pro-inflammatory cytokines, IFN-γ and IL-17 from PLNC restimulated with MOG ex vivo (Figures 2H,I). Lymph node cells obtained from non-immunized counterparts did not produce detectable amounts of IFN-γ and IL-17 in response to MOG. GH treatment did not have a significant effect on mRNA expression of IL-10 or TGF-β (Figures 2J,K).

### GH influence on biochemical profile during EAE

To assess the systemic effects of EAE induction and GH treatment, a comprehensive serum biochemical profile was conducted at the peak and end of EAE (Supplementary Table S1 – Data Sheet 3), for the following experimental groups: peak of EAE in non-treated (Ep) and GH-treated rats (EpGH), end of EAE in non-treated (Ee) and GH-treated rats (EeGH), and non-immunized—control rats (C). Serum levels of key pro- and anti-inflammatory cytokines, including IL-10, TNF-α, IFN-γ, and IL-17, were also measured across all experimental groups; however, their concentrations remained below the limit of detection (data not shown), suggesting that the inflammatory response was primarily localized within the CNS rather than presenting as a marked systemic cytokine storm. Despite the absence of detectable serum cytokines, the systemic metabolic and tissue-damage response was highly pronounced during the peak of EAE. EAE induction resulted in a marked increase in serum ALT and AST levels, along with a substantial elevation of CK (nearly two-fold) and LDH (two-fold) compared to the control group, indicating severe cellular stress, muscle wasting, and systemic tissue damage during the acute phase of the disease. GH administration at the peak of EAE significantly mitigated this systemic damage. Treatment with GH reduced the levels of ALT, AST, CK, and LDH compared to the untreated Ep group, bringing these parameters closer to baseline control values. For other parameters, a significant increase in GGT and Crea levels was observed at the peak of EAE, which also remained elevated in the EpGH group. At the end of the disease (Ee and EeGH groups), ALT, AST, CK, and LDH levels stabilised, showing no significant differences compared to the control, although a persistent elevation in Crea and ALP was noted in both groups at the end of EAE. No major or biologically relevant fluctuations were observed in lipid status (Chol, Trig) or electrolyte levels (Ca, Mg) across the groups (Supplementary Table S1).

### GH modulates microglia/ macrophages activation phenotype

Next, we investigated the effect of GH on microglia/ macrophages-associated changes in EAE. To this end, we analyzed the expression patterns of markers related to microglia/ macrophages activation and inflammatory polarization in the spinal cord of EAE and EAE + GH rats at two time points: the peak and end of EAE. We first examined *Cd68*, *Itgam*, and *Cx3cr1*, markers associated with microglia/ macrophages activation and immune cell recruitment. As expected, *Cd68* showed low basal expression in non-immunized rats and was significantly upregulated at the peak of EAE, whereas GH treatment markedly attenuated this increase. A similar pattern was observed for *Itgam*, which was strongly induced at the peak of disease and significantly reduced by GH treatment at this stage. Likewise, *Cx3cr1* expression was elevated at the peak of EAE and partially reduced by GH, while no significant differences were observed at the end stage of the disease (Figures 3A–C). We next analyzed *Aif1*, a general marker of microglia/macrophages presence. *Aif1* expression was low in control animals and significantly increased at the peak of EAE. This increase was markedly reduced by GH treatment. By the end of EAE, *Aif1* levels declined compared to the peak, and GH had no significant effect at this time point (Figure 3D). The expression of *Mrc1*, a marker associated with anti-inflammatory microglia/macrophages phenotypes, showed a modest increase at the peak of EAE, which was further enhanced by GH treatment. However, at the end stage of the disease, *Mrc1* expression returned to low levels and was not significantly influenced by GH (Figure 3E). Finally, we assessed genes associated with inflammatory effector functions. *Nos2* expression was markedly increased at the peak of EAE and significantly reduced by GH treatment, following the overall inflammatory pattern observed for activation markers. In contrast, *Arg1* expression remained low across all groups and was not significantly affected by immunization, disease stage, or GH treatment (Figures 3F–G).

**Figure 3:**
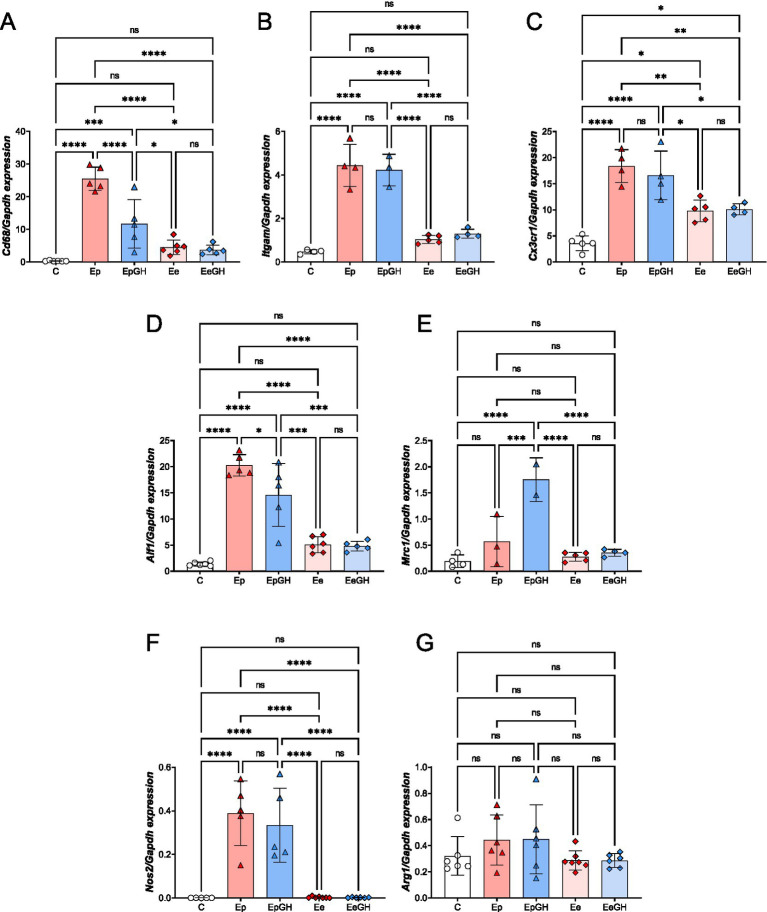
Effect of GH on expression of microglia/macrophages activation-related genes in EAE. qPCR was performed on samples obtained from non-immunized rats (C), untreated (Ep) or GH-treated (EpGH) rats at the peak of the disease, or untreated (Ee) or GH-treated (EeGH) rats after recovery from the disease. Relative expression levels of mRNA for *Cd68* **(A)**, *Itgam* **(B)**, *Cx3cr1* **(C)**, *Aif1* **(D)**, *Mrc1* **(E)**, *Nos2* **(F)**, an*d Arg1* **(G)** were determined. Data are presented as mean + SD (*n* = 5/6). ^*^p < 0.05, ^**^p < 0.01, ^****^p < 0.001, ^***^p < 0.0001, ns, not significant.

### GH reduces ED1^+^ microglia/macrophages activation and preserves MBP during EAE peak

During the peak of EAE, a highly dense and clustered red fluorescence signal corresponding to ED1-positive reactive microglia/macrophages was observed. This intense phagocytic/inflammatory signal coincided with a marked reduction in green MBP fluorescence. In contrast, spinal cord sections from the GH-treated group showed a pronounced attenuation of ED1 immunoreactivity, characterized by reduced punctate intensity and decreased cellular clustering, predominantly localized to perivascular regions (Figure 4A). This reduction in microglia/macrophages reactivity was accompanied by preservation of a more continuous, homogeneous MBP signal. Consistent with these observations, the percentage area occupied by ED1-positive structures was significantly increased at the peak of EAE, whereas GH treatment markedly reduced ED1-positive area (Figure 4B). At the end stage of the disease, both EAE and GH-treated groups showed negligible ED1 immunoreactivity (Figures 4B,C; Supplementary Figure 2 – Data Sheet 2). Quantitative analysis further demonstrated a significant increase in both IntDen and CTCF at the peak of EAE compared to controls, reflecting robust microglia/macrophage activation and macrophage infiltration. In contrast, GH administration at peak EAE significantly reduced both parameters compared to untreated EAE animals (Figure 4C). No significant differences were observed at the end stage between groups.

**Figure 4:**
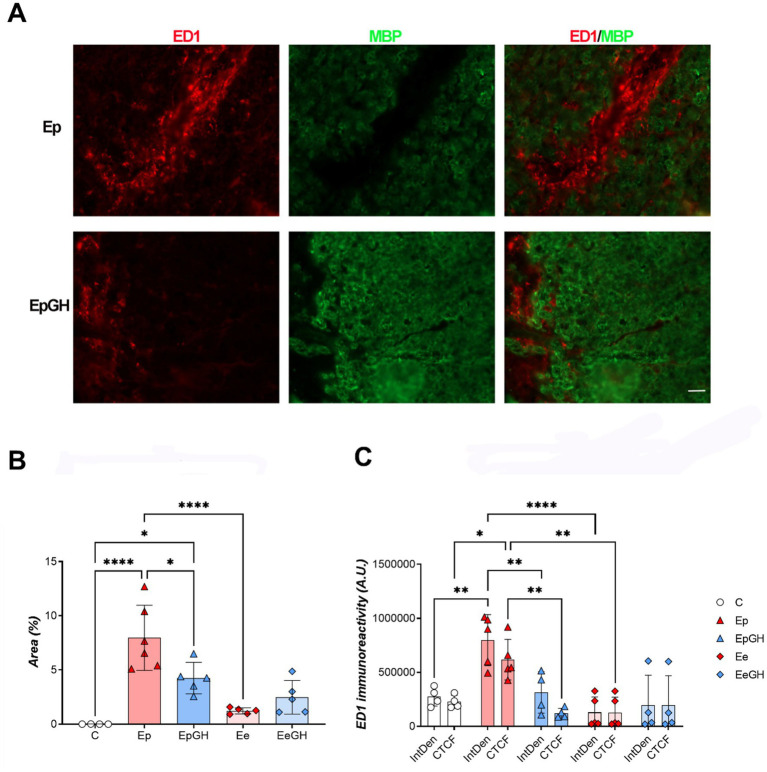
Growth hormone reduces microglia/macrophages phagocytic activation and preserves myelin integrity following EAE. **(A)** Representative immunofluorescence images of spinal cord sections (16 μm sections) stained for ED1 (red; lysosomal marker of phagocytic microglia/macrophages), reflecting the characteristic punctate staining, and myelin basic protein (MBP, green), and merged signals, from the peak of EAE (Ep) and the GH-treated EAE group (EpGH). Scale bar: 20 μm. **(B)** Quantification of ED1-positive area (%). **(C)** Densitometric analysis of ED1 expression using Integrated Density (IntDen) and Corrected Total Cell Fluorescence (CTCF), expressed in arbitrary units. Data are presented as mean ± SD. ^*^p < 0.05, ^**^p < 0.01, ^****^p < 0.001, ^***^p < 0.0001, ns, not significant.

### GH modulates microglia/macrophage morphology during EAE progression

Finally, the effect of GH on microglia/macrophage morphology in EAE was determined. Representative immunofluorescence micrographs of IBA1^+^ cells revealed noticeable disease stage—dependent changes in its number and morphology (Figure 5). In control animals, microglia/macrophage displayed a typical ramified morphology with small cell bodies and long, thin, highly branched processes (Figure 5A). At the peak of EAE, a markedly increased number of IBA1^+^ cells was observed, accompanied by pronounced morphological heterogeneity dominated by amoeboid and poorly ramified phenotypes, characterized by enlarged somata and retracted processes (Figure 5B). GH treatment at the peak of disease attenuated these morphological alterations, as microglia/macrophage exhibited more elongated and branched processes and reduced compactness compared to untreated EAE animals, although their morphology remained distinct from controls (Figure 5C). In parallel, GH treatment significantly reduced the number of IBA1^+^ cells compared to the peak of EAE (Figure 5F). At the end of disease, the number of IBA1^+^ cells was reduced compared to the peak of EAE, and microglia/macrophage morphology partially recovered, with increased process length and branching, yet without full restoration of the control phenotype (Figure 5D). In GH-treated animals at the end of disease, microglia/macrophage number was further reduced compared to untreated EAE animals, and those cells displayed additional increases in process extension and branching complexity, resulting in a more ramified appearance (Figure 5E). For each experimental group, a representative IBA1^+^ cell is indicated by a rectangle, with corresponding enlarged insets showing the skeletonized cell (upper inset) (a, b, c, d, e) and the outline used for fractal analysis (lower inset) (a’, b’, c’, d’, e’, f’), illustrating group-specific differences in branching architecture and cell complexity.

**Figure 5:**
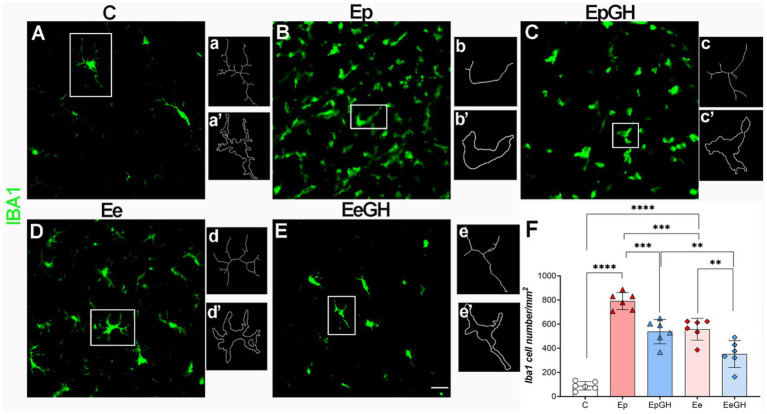
Effect of GH on microglia/macrophage number and morphology in EAE. Representative fluorescence microscopy images of IBA1^+^ cells on spinal cord sections (50 μm sections) stained with IBA1 (green). Spinal cord sections were obtained at the peak of disease from non-immunized—C **(A)**, untreated—Ep **(B)**, or GH-treated animals—EpGH **(C)**, or at the end of disease from untreated—Ee **(D)**, or GH-treated—EeGH animals **(E)**. Binary isolated and skeletonized microglia/macrophage **(a–e)**, isolated microglia/macrophage converted to outlines for fractal analysis **(a′–e′)**. Scale bar referring to **A–E**: 20 μm. Graph **(F)** represents the number of microglia/macrophage cells in spinal cord during EAE with or without GH treatment, quantified from six different animals per group. Data are presented as mean + SD. ^*^p < 0.05, ^**^p < 0.01, ^****^p < 0.001, ^***^p < 0.0001, ns, not significant.

To quantitatively evaluate the effect of GH on microglia/macrophage morphometry during EAE, we analyzed their cell size and shape and performed single-cell skeleton and fractal analyses. Morphometric analysis demonstrated that microglia/macrophage at the peak of disease exhibited a significantly smaller cell area and perimeter compared to control animals and those cells at the end of disease (Figures 6A,B). These changes were consistent with the immunofluorescence images and reflected process retraction and acquisition of a compact, activated phenotype, further supported by significantly increased circularity, roundness and solidity values at the peak of EAE (Figures 6C–E). GH treatment increased microglia/macrophage area and perimeter at the peak of disease compared to untreated EAE animals [an unpaired Welch’s *t*-test demonstrated a highly significant difference between Ep and EpGH groups (*t*(7.9) = 9.65, *p* < 0.0001)]. The mean value in the EpGH group (172.9 ± 27.0) was substantially higher than in the Ep group (75.8 ± 9.6), with a mean difference of 97.1 (95% CI: 73.6–120.6). The effect size was very large (Cohen’s *d* = 3.94). An *F*-test indicated significantly different variances between groups [*F*(5,5) = 8.0, *p* = 0.02], while significantly reducing circularity, roundness and solidity, indicative of partial morphological normalization. At the end of disease, microglia/macrophage area and perimeter were increased in both EAE and GH-treated groups compared to controls; however, differences between untreated and GH-treated animals at this stage did not reach statistical significance, as with solidity, roundness and circularity (Figures 6A–E).

**Figure 6:**
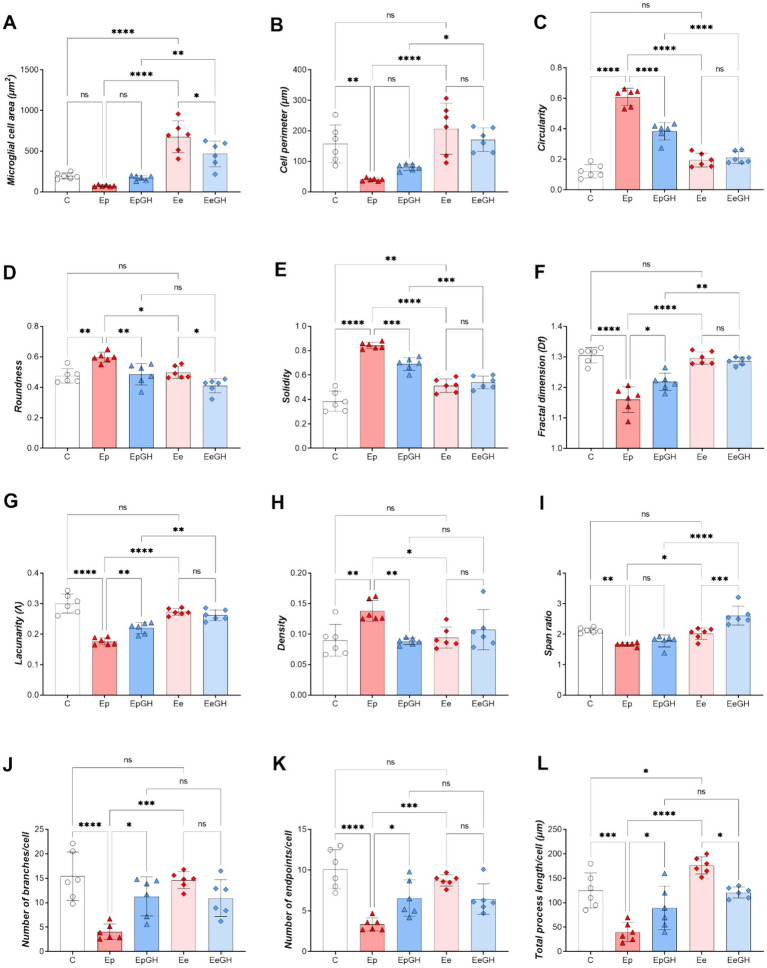
Effect of GH on microglia/macrophage morphology in EAE. Graphs **(A–E)** represent cell size and shape: Area (μm^2^), perimeter (μm), circularity, roundness, and solidity. Graphs **(F–I)** represent fractal analysis of microglia/macrophage complexity: fractal dimension, lacunarity, span ratio, and density **(J–L)** represent skeleton analysis: number of branches, number of endpoints, total process length (μm). Data are presented as mean + SD from 6 rats per group, in total 120–150 cells/group. ^*^p < 0.05, ^**^p < 0.01, ^****^p < 0.001, ^***^p < 0.0001, ns, not significant.

Fractal analysis demonstrates a significant loss of microglia/macrophage morphological complexity at the peak of EAE (Figures 6F–I). Fractal dimension, lacunarity, and span density were significantly reduced at this stage compared to controls and microglia/macrophage at the end of disease, while cell density was significantly increased, consistent with a compact, amoeboid phenotype. GH treatment partially restored microglia/macrophage complexity at the peak of disease, resulting in significantly higher fractal dimension and lacunarity, together with a significant reduction in density compared to untreated EAE animals. At the end of disease, GH treatment did not significantly alter fractal dimension, lacunarity or density relative to untreated EAE animals; however, span ratio was significantly increased, indicating sustained modulation of microglia/macrophage structural organization.

Single-cell skeleton analysis corroborated the fractal analysis findings by further revealing marked alterations in microglial branching architecture across disease stages (Figures 6J–L). At the peak of EAE, IBA1^+^ cells exhibited a significant reduction in the number of branches, number of endpoints, and total branch length compared to control animals. GH treatment significantly increased all skeleton parameters at the peak of disease relative to untreated EAE animals, consistent with enhanced process extension. At the end of the disease, untreated EAE animals showed a partial recovery of branching parameters; however, GH treatment did not affect the number of branches or endpoints. In contrast, total branch process length was significantly reduced in GH-treated rats compared to untreated EAE animals at the end stage of EAE.

In addition to observing morphometric changes in microglia/macrophage during EAE, we performed quantitative colocalization analysis between IBA1 and iNOS or ARG1 to further characterize the functional state of microglia/macrophages (Figure 7). Colocalization between IBA1 and iNOS was assessed using Pearson’s correlation coefficient (*P*) and Manders’ overlap coefficients (M1 and M2), which provide complementary information on signal covariance and spatial overlap, respectively. At the peak of EAE, both Pearson’s correlation coefficient and Manders’ M2 coefficient for IBA1/iNOS were significantly increased, whereas both parameters were reduced at the end of the disease (Figure 7). The increase in Manders’ M2 indicates that a larger fraction of the iNOS signal is localized within IBA1^+^ cells during peak neuroinflammation, consistent with enhanced microglia/ macrophages pro-inflammatory activity. The subsequent decrease in M2 at disease resolution reflects reduced microglia/ macrophages association with iNOS. In contrast, Manders’ M1 coefficient remained unchanged across all experimental groups, indicating that the proportion of the total IBA1 signal overlapping with iNOS-positive structures did not change. This suggests that iNOS expression is restricted to a subset of microglia/macrophages and that disease progression primarily affects the distribution and intensity of iNOS within microglia/macrophages rather than the proportion of microglia/macrophages expressing iNOS. GH treatment significantly reduced both Pearson’s correlation coefficient and Manders’ M2 coefficient at the peak of EAE compared with untreated animals, indicating decreased localization and reduced covariance of iNOS within microglia/macrophages during the inflammatory phase. Notably, at the end of the disease, both Pearson’s correlation and Manders’ M2 were significantly increased in GH-treated animals compared with untreated EAE animals, suggesting enhanced microglia/macrophages association of iNOS during the recovery phase. Importantly, Manders’ M1 coefficient remained unchanged across all disease stages and treatments, indicating that GH modulates iNOS distribution and regulation within microglia/macrophages, rather than altering the overall proportion of iNOS-associated microglia/macrophages. Colocalization analysis between IBA1 and ARG1 revealed a different pattern. Pearson’s correlation coefficient was significantly increased at the peak of EAE in both untreated and GH-treated animals, indicating increased covariance of IBA1 and ARG1 signals during acute inflammation. However, no significant changes were observed in Manders’ M1 or M2 coefficients across disease stages or treatments. This indicates that although the intensity relationship between IBA1 and ARG1 changes during EAE, the spatial overlap between ARG1 and microglia/macrophages remains stable, suggesting modulation of microglia/macrophages activation state rather than a shift in microglia/macrophages phenotypic composition.

**Figure 7:**
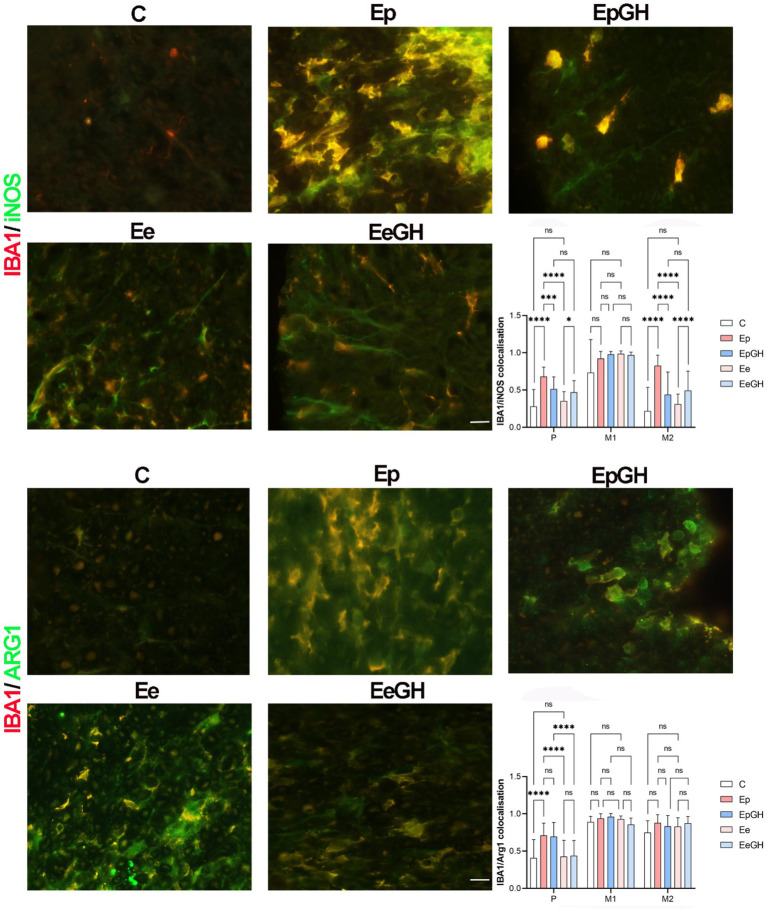
Effects of GH on microglia/ macrophages phenotype in EAE. Representative fluorescence microscopy images of microglia/ macrophages on spinal cord sections (16 μm sections) stained with IBA1 (red), iNOS (green), ARG1 (green). Non-immunized rats (C), untreated (Ep) or GH-treated (EpGH) rats at the peak of the disease, or untreated (Ee) or GH-treated (EeGH) rats after recovery from the disease. Colocalization IBA1/iNOS and IBA1/ARG1 analysis graphs: *P*—Pearson’s correlation coefficient, M1—M1 Manders’ colocalization coefficient, M2—M2 Manders’ colocalization coefficient. Data are mean ± SD; *n* = 3 per group. Scale bars: 10 μm. ^*^p < 0.05, ^**^p < 0.01, ^****^p < 0.001, ^***^p < 0.0001, ns, not significant.

## Discussion

Our study demonstrates that GH exerts robust anti-inflammatory and antioxidant effects on activated BV2 microglia. In addition, GH treatment modulates both peripheral T cell activation, as well as central microglia/macrophages morphology and function during EAE. Specifically, GH treatment delayed disease onset, reduced pro-inflammatory cytokine production by peripheral lymphocytes, and decreased microglia/macrophages numbers in the spinal cord at the peak of disease. Importantly, the remaining microglia/macrophages exhibited improved morphometric features, despite the absence of a complete shift in classical M1/M2 gene expression markers. However, given the multi-faceted systemic and central pathogenesis of EAE, it remains to be fully elucidated whether these *in vivo* alterations stem entirely from a direct action of GH on microglia/macrophages, or are secondary to the GH-mediated modulation of peripheral immune responses and cell trafficking into the CNS.

First, we evaluated the effect of GH treatment in LPS-activated BV2 microglia, where this treatment significantly reduced NO and TNF production. Similar anti-inflammatory effects of GH have also been reported in the SIM-A9 microglial cell line, where GH treatment similarly suppressed pro-inflammatory mediator release following LPS stimulation (Balderas-Márquez et al., 2025). In our study, GH also reduced soma hypertrophy and counteracted cytoskeletal remodeling, consistent with anti-inflammatory effects (Soler Palacios and Nieto, 2020). Notably, higher GH doses have been reported to exert pro-inflammatory effects in macrophages (Smith et al., 2000; Schneider et al., 2019), emphasizing the importance of dose and cell-type specificity. The observed anti-inflammatory effects in our study may therefore be due to the lower GH dose and cell-type specific differences, including GHR expression (Taciak et al., 2018). Beyond inflammation, GH also exerted marked antioxidant effects. While GH deficiency is associated with oxidative stress, GH therapy has the potential to restore redox balance in several biological systems (Mancini et al., 2020; Kościuszko et al., 2025). Here, we demonstrate for the first time that GH suppresses LPS-induced oxidative stress in BV2 microglia, reducing superoxide anion levels and lipid peroxidation, while enhancing the antioxidant defense system, including catalase, reduced glutathione, and glutathione reductase. The modest reduction in GPx activity suggests selective redox modulation rather than uniform upregulation, consistent with adaptive antioxidant responses reported in ageing and stress models (Csiszár et al., 2016; Esimo et al., 2019). While these *in vitro* data provide valuable mechanistic insights, we acknowledge that the BV2 cell line and LPS stimulation do not fully capture the functional complexity of primary microglia or the multi-faceted pathogenesis of EAE. Nevertheless, this reductionist system served as a highly reproducible tool to isolate the direct effects of GH on microglial activation, independent of systemic *in vivo* interactions. Therefore, these findings should be interpreted strictly as complementary mechanistic support to our principal in vivo results.

These results led us to investigate the effect of GH treatment on EAE disease onset and progression. Therapeutic strategies that limit lymphocyte infiltration across the blood–brain barrier or promote microglia/macrophages ramification and an anti-inflammatory phenotype are increasingly recognized as effective approaches to mitigate pathology in MS/EAE (Fischer et al., 2021; Mahmood and Miron, 2022; Nan et al., 2024). Previous studies have shown that GH treatment can prevent or ameliorate autoimmune conditions, including type I diabetes and collagen-induced arthritis (Villares et al., 2013, 2018). Moreover, preliminary clinical observations suggest a potential role for GH in promoting remyelination in MS patients (Stoppe et al., 2014), an effect also reported in cuprizone-induced demyelination (Bekheet and Sonbol, 2023).

We have shown that GH significantly reduced IL-17 and IFN-γ production by lymphocytes isolated from popliteal lymph nodes, without affecting total lymphocyte numbers and mRNA expression of IL-10 and TGF-β, indicating a selective immunomodulatory effect on pathogenic T cell responses. Although our transcriptional data suggest no immediate effect of GH on these anti-inflammatory markers, the absence of protein-level quantification, such as ELISA or multiplex assays, remains a limitation that should be addressed in future studies. Suppression of IFN-γ and IL-17 is consistent with our previous reports linking Th1 and Th17 inhibition to reduced EAE severity in DA rats (Momcilović et al., 2008; Miljković et al., 2009, 2015). GH signaling has been shown to influence T cell differentiation in autoimmune contexts (Arellano et al., 2024), and pharmacological modulation of GHRH receptor signaling can attenuate Th17-mediated neuroinflammation (Du et al., 2022, 2023). These peripheral immunological effects are consistent with the delayed onset of clinical disease observed in GH-treated animals. Delaying disease onset and shortening paralysis duration which are clinically relevant endpoints in MS is of utmost interest indicating GH potential to preserve neurological reserve and to limit irreversible axonal damage during early neuroinflammatory episodes.

Notably, systematic profiling of serum inflammatory mediators (including TNF, IL-1β, IFN-ɣ and IL-10) revealed that these cytokines remained strictly below the limit of detection across all experimental groups, a finding that aligns with the established paradigm that EAE-driven neuroinflammation is highly compartmentalized and restricted to the CNS microenvironment, where peripheral cytokine dilution and rapid clearance typically uncouple systemic serum profiles from local tissue pathology (Gold et al., 2006; Constantinescu et al., 2011). However, beyond these localized immunomodulatory effects, the systemic impact of GH treatment significantly mitigated the systemic metabolic burden and tissue damage driven by EAE. While the peak of disease was marked by severe systemic stress and muscle wasting secondary to neurological paralysis (Luque et al., 2015), evidenced by dramatic spikes in serum creatinine kinase and LDH (Baird et al., 2012; Shipman et al., 2024), GH administration effectively countered this damage by significantly lowering these parameters. This demonstrates a potent hepatoprotective effect and a clear reduction in peripheral muscle injury during peak neuroinflammation, which aligns with the known anabolic and cytoprotective properties of GH under severe inflammatory or catabolic stress (Velloso and Donato, 2024; Moller et al., 2009).

Within the central nervous system, GH treatment significantly reduced the transcriptional levels of *Aif1* and *Cd68* at the peak of EAE, markers associated with phagocytic activity and microglia/ macrophages activation, respectively (Jurga et al., 2020), which is consistent with our previous findings (Jakovljevic et al., 2019). The suppression of *Cd68* transcription was further confirmed at the protein level, as immunohistochemical analysis revealed a significant reduction in ED1 phagocytic cells, within demyelinating lesions. Notably, these phagocytic cells were predominantly restricted to the perivascular space, with very few cells infiltrating the parenchyma. This distinct spatial distribution implies that GH treatment actively hinders or delays the extravasation and parenchymal infiltration of these peripheral myeloid cells during acute neuroinflammation, thereby protecting the central nervous system from aggressive phagocytic damage, a phenomenon that contrasts with the massive, widespread parenchymal infiltration of ED1 cells typically observed at the peak of EAE (Trifunović et al., 2015). To further support this, our histological analysis using hematoxylin staining corroborated a marked reduction in inflammatory cell infiltrates within the spinal cord tissue of GH-treated animals. Furthermore, GH-treated animals at peak EAE exhibited a significant downregulation of *Itgam* (encoding CD11b) and *Cx3cr1*, indicating a restricted microglia/macrophages activation and chemotactic signaling. In contrast, Nos2 and Arg1 expression remained unchanged, while GH treatment selectively upregulated the transcription of *Mrc1* (encoding CD206) at the peak of disease. This stands in contrast to certain models of spinal cord injury where GH administration has been reported to decrease overall *Mrc1* expression alongside pro-inflammatory markers (Martínez-Moreno et al., 2023; Balderas-Márquez et al., 2025). This discrepancy is likely due to differences in cellular dynamics and infiltration patterns within the tissue. Because bulk tissue qPCR reflects relative transcript abundance, the apparent increase in *Mrc1* expression in our model should be interpreted in light of the significantly reduced cellular density and limited parenchymal infiltration of ED1 phagocytes observed under GH treatment. By drastically restricting the influx of aggressive, pro-inflammatory peripheral myeloid cells into the CNS parenchyma, GH alters the cellular composition of the lesion site. Consequently, the relative proportion of resident, homeostatic or regulatory myeloid subsets that constitutively express *Mrc1* becomes enriched. Thus, the increase in *Mrc1* transcription may reflect a preservation of the tissue’s endogenous regulatory and reparative reserve rather than a classical, direct phenotypic polarization of all myeloid cells towards an alternative anti-inflammatory state. Morphometric analysis revealed that microglia/macrophages at the peak of disease displayed a typical neuroinflammatory phenotype (Green et al., 2022), with a pronounced amoeboid shape, characterized by reduced cell area and perimeter, increased circularity, roundness and solidity, diminished branching complexity, and shortened total branch length, reflecting extensive process retraction and cellular compaction. Fractal analysis further confirmed a simplified architecture, with reduced fractal dimension, lacunarity, and span ratio, accompanied by increased cell density, features consistent with a hyperactivated, pro-inflammatory microglia/macrophages state (Jakovljevic et al., 2019; Young and Morrison, 2018; Bishnoi and Bordt, 2025), (Fernández-Arjona et al., 2017). GH treatment at the peak of EAE partially reversed these alterations, increasing cell area and perimeter, branch number and endpoints, total branch length, fractal dimension, lacunarity, and span ratio, while reducing microglia/macrophages density, circularity, roundness and solidity. GH treatment significantly reduced the colocalization of iNOS with IBA1, indicating a suppression of pro-inflammatory microglia/macrophages activity. In contrast, GH did not alter Arginase-1 colocalization with IBA1, suggesting that GH does not promote a classical alternative microglia/macrophages phenotype, but rather selectively attenuates detrimental inflammatory signaling. Collectively, these changes indicate a shift towards a more ramified, surveillant phenotype, further supported by reduced iNOS/IBA1 colocalization, reflecting diminished localization of pro-inflammatory enzymes within microglia/macrophages, as previously noted in spinal cord injury model (Martínez-Moreno et al., 2023).

At the end of the disease, microglia/macrophages exhibited a partially recovered phenotype characterized by hypertrophy combined with increased ramification, suggesting a transition towards homeostatic surveillance. Fractal parameters and branching complexity were increased relative to the disease peak, while density was reduced, indicating structural normalization. GH treatment at this stage further promoted ramification, although differences between treated and untreated groups were less pronounced, likely reflecting intrinsic recovery mechanisms. Similar GH-mediated effects on microglia/macrophages branching complexity and fractal organization have been reported in the retina following optic nerve crush injury, where GH treatment promoted microglia/macrophages ramification and restoration of complex fractal patterns (Balderas-Márquez et al., 2025). The modest enhancement of branching complexity under GH treatment at this stage, suggests that the primary efficacy of GH treatment lies in restraining early neurotoxic activation rather than overriding late-phase recovery. These stage-dependent effects underscore the importance of therapeutic timing when targeting microglia/macrophages function in neuroinflammatory disease.

Together, these results demonstrate that GH acts in a clear, stage-dependent manner. During acute neuroinflammation, GH treatment is associated with a reduction in neurotoxic microglia/macrophages markers, while in the later phases of the disease, it correlates with microglia/macrophages re-ramification and homeostatic surveillance, without hindering ongoing reparative processes. A limitation of the present study is the use of recombinant human GH (rhGH), chosen for practical and translational reasons and widely used in rodent models. Additionally, the absence of a healthy control group treated with GH (Control + GH) represents another limitation of our experimental design. Although previous studies have shown that intravenous administration of GH does not cross the intact blood-spinal cord barrier, spinal cord injury markedly increases its permeability (Mustafa et al., 1995). Hence, the lack of a Control + GH group precludes a definitive delineation between disease-specific therapeutic mechanisms and potential baseline physiological or immunomodulatory actions of GH in an intact system. Future investigations incorporating this control group, alongside studies directly comparing sex-specific responses and varying GH dosages, will be essential to fully elucidate the baseline effects of GH, refine therapeutic strategies, and optimize translational relevance. Importantly, the lack of direct, localized cytokine profiling within the CNS tissue or cerebrospinal fluid represents a significant caveat. While we assessed key proinflammatory cytokines at the protein level *ex vivo* and evaluated microglia/ macrophages morphology extensively, the actual protein levels of the pro-inflammatory cytokine milieu within the CNS compartment remain uncharacterized. Although our data demonstrates a clear shift in microglia/ macrophages activation following GH treatment, it remains to be fully elucidated whether these changes are entirely due to direct GH signaling on microglia/macrophages or are, in part, secondary to the overall reduction in peripheral immune cell infiltration, as evidenced by the attenuated inflammatory infiltrates. Further studies utilizing microglia/macrophages cell-specific deletions would be required to completely dissect these pathways.

In conclusion, our results suggest that GH is unlikely to be effective as a stand-alone therapy, but may be more beneficial as an adjunct or early intervention strategy. Furthermore, our findings indicate that GH acts as a modulator of early neuroimmune interactions rather than as a classical immunosuppressant, supporting its potential as a timing-sensitive adjunctive strategy in neuroinflammatory disease. Nevertheless, further studies on the therapeutic use of GH in MS are warranted.

## Data Availability

The original contributions presented in the study are included in the article/Supplementary material, further inquiries can be directed to the corresponding author.
