# Supplementary material for: Growth hormone restrains inflammatory activity of microglia/macrophages in CNS autoimmunity
Source: Front Cell Neurosci. 2026 Aug 4;20:1828468. doi: 10.3389/fncel.2026.1828468 (PMC13481192; doi:10.3389/fncel.2026.1828468)
Supplement: Supplementary file 1 [file Supplementary_Figure_1.pdf]

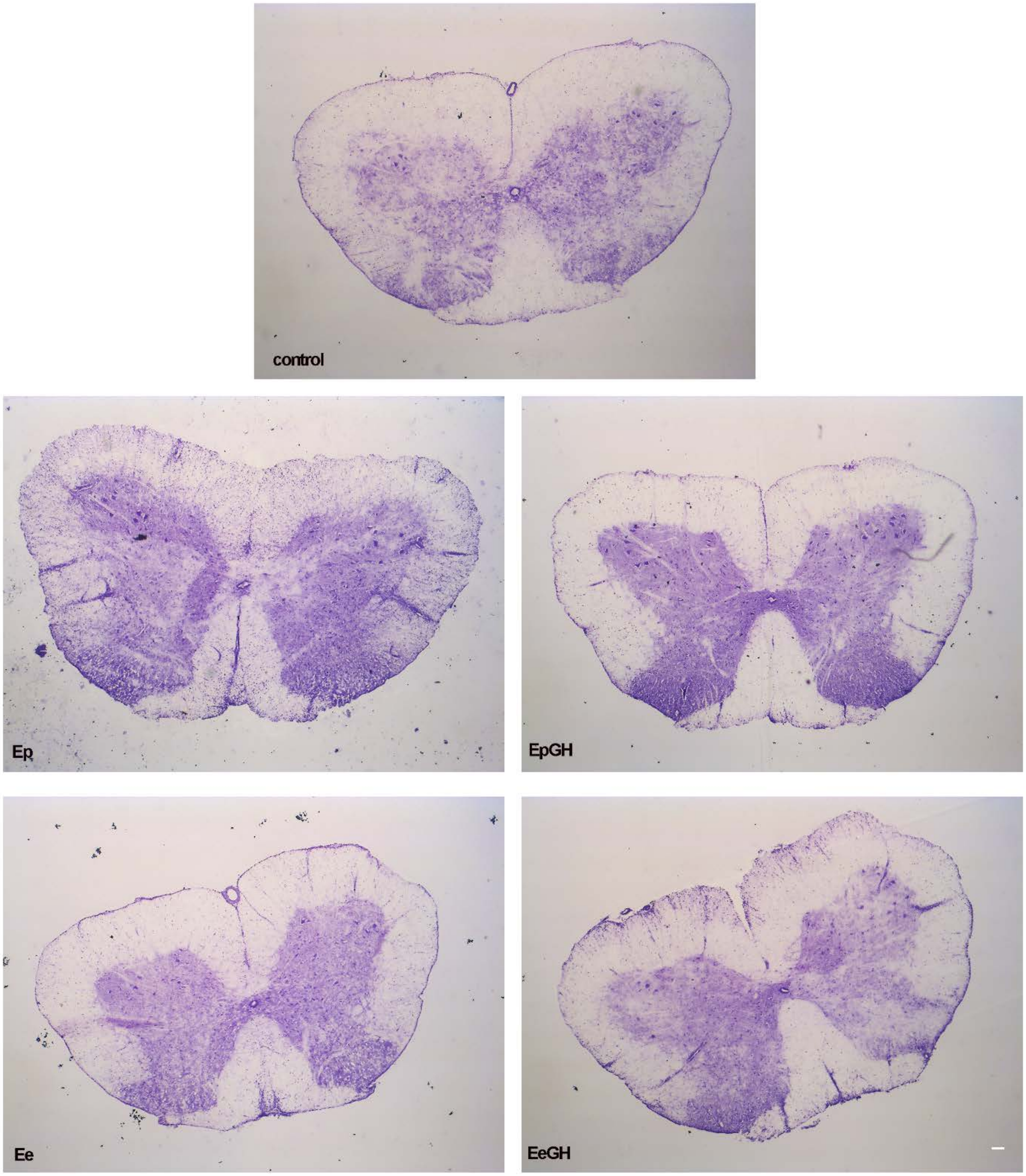

Supplementary Figure 1. Growth hormone attenuates spinal cord infiltration during EAE. Representative hematoxylin-stained sections of the spinal cord are shown for control rats, animals at the peak of EAE (Ep), GH-treated rats at disease peak (EpGH), untreated rats at the end of disease (Ee), and GH-treated rats at disease resolution (EeGH). Scale bar: 100 µm.
