# Supplementary material for: Growth hormone restrains inflammatory activity of microglia/macrophages in CNS autoimmunity
Source: Front Cell Neurosci. 2026 Aug 4;20:1828468. doi: 10.3389/fncel.2026.1828468 (PMC13481192; doi:10.3389/fncel.2026.1828468)
Supplement: Supplementary file 2 [file Supplementary_Figure_2.pdf]

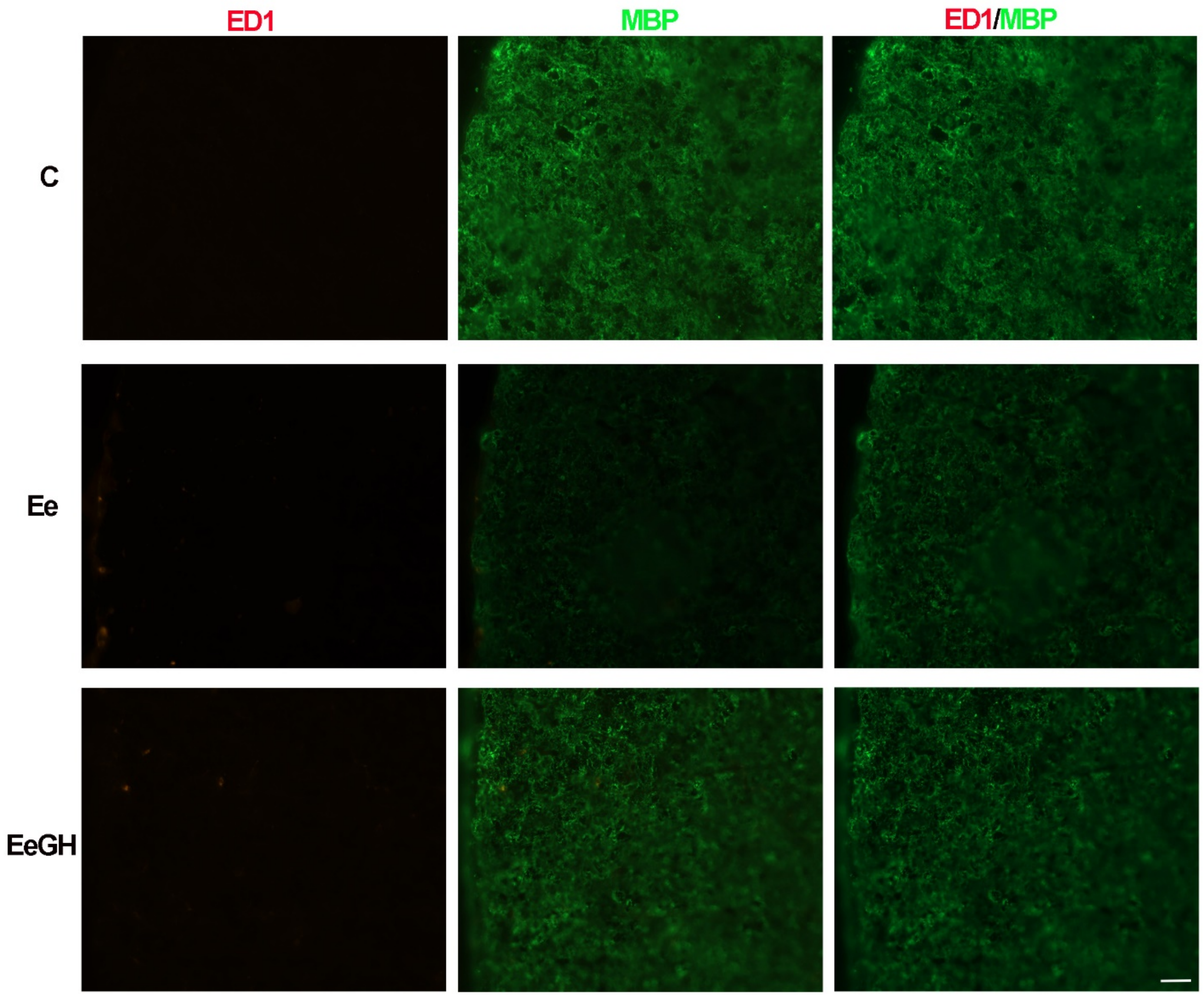

Supplementary Figure 2: Growth hormone reduces microglial phagocytic activation and preserves myelin integrity following EAE. Representative immunofluorescence images of spinal cord sections stained for ED1 (red; lysosomal marker of phagocytic microglia/macrophages) and myelin basic protein (MBP, green), and merged signals, from the control (C), end of disease (Ee) and GH treated EAE group (EeGH). Scale bar: 20 µm.
