# Supplementary material for: Growth hormone restrains inflammatory activity of microglia/macrophages in CNS autoimmunity
Source: Front Cell Neurosci. 2026 Aug 4;20:1828468. doi: 10.3389/fncel.2026.1828468 (PMC13481192; doi:10.3389/fncel.2026.1828468)
Supplement: Supplementary file 3 [file Supplementary_Figure_3.pdf]

Supplementary Table 1: Impact of growth hormone administration on systemic metabolic and biochemical parameters in EAE.

	**C**	**Ep**	**EpGH**	**Ee**	**EeGH**
Albumin	33.15 ± 0.24	29.82 ± 1.16 *	29.15 ± 0.74 **	32.47 ± 0.40	33.55 ± 0.37
ALP	217.43 ± 6.15	131.28 ± 12.95 ***	127.20 ± 11.96 ***	282.40 ± 11.61 **	248.80 ± 11.02 *
**ALT**	**88.87 ± 3.60**	**99.60 ± 10.05**	**67.00 ± 6.31**$^{*\#}$	**99.10 ± 4.87**	**97.75 ± 4.71**
**AST**	**175.92 ± 10.53**	**271.42 ± 26.91** ***	**162.22 ± 16.76** $^{\#}$	**184.65 ± 21.81**	**179.87 ± 25.73**
Bil-T	2.83 ± 0.23	1.60 ± 0.21 ***	1.55 ± 0.14 ***	1.45 ± 0.08 ***	1.65 ± 0.07 ***
Ca	2.61 ± 0.04	2.65 ± 0.06	2.78 ± 0.05 *	2.80 ± 0.09 *	2.74 ± 0.06
Chol	2.33 ± 0.05	2.59 ± 0.20	2.71 ± 0.13 *	2.66 ± 0.13 *	2.52 ± 0.14
**CK**	**647.17 ± 81.33**	**1142.83 ± 222.96****	**570.33 ± 75.16** $^{\#}$	**556.67 ± 127.22**	**420.50 ±134.48**
Crea	30.83 ± 0.87	60.83 ± 2.09 ***	59.67 ± 4.74 ***	58.00 ± 1.83 ***	60.67 ± 1.50 ***
GGT	3.37 ± 0.42	7.43 ± 1.25 **	7.08 ± 0.69 **	5.67 ± 0.60 *	4.75 ± 0.70
Glu	9.62 ± 0.57	8.25 ± 0.86	9.37 ± 0.50	7.77 ± 0.44 *	8.23 ± 0.35
**LDH**	**1097.83 ± 129.28**	**2187.50 ± 192.98** ***	**1211.50 ± 175.35**$^{\#}$	**1071.67 ± 204.53**	**897.67 ±229.97**
Prot-T	63.55 ± 0.94	68.00 ± 0.88 *	69.28 ± 1.63 **	64.65 ± 0.94	67.38 ± 0.93
Trig	0.49 ± 0.07	0.37 ± 0.03	0.41 ± 0.03	0.57 ± 0.03 *	0.44 ± 0.06
Urea	8.18 ± 0.41	7.38 ± 0.45	7.95 ± 0.30	7.45 ± 0.20	8.22 ± 0.49
BHB	0.21 ± 0.03	0.48 ± 0.13 *	0.35 ± 0.05	0.18 ± 0.01	0.17 ± 0.01
Mg	0.77 ± 0.01	0.79 ± 0.02	0.82 ± 0.01 *	0.77 ± 0.02	0.77 ± 0.01
TBA	21.23 ± 3.41	14.52 ± 4.26	9.93 ± 3.77	39.32 ± 13.93 *	44.94 ± 17.13 *

(* $p < 0.05$, * p < 0.01, *** p < 0.001 vs C)
(# $p < 0.05$ Ep vs EpGH)
